# COVID-19 vaccination coverage by company size and the effects of workplace vaccination program in Japan: a cohort study

**DOI:** 10.1265/ehpm.22-00091

**Published:** 2022-07-01

**Authors:** Koji Mori, Takahiro Mori, Tomohisa Nagata, Hajime Ando, Ayako Hino, Seiichiro Tateishi, Mayumi Tsuji, Keiji Muramatsu, Yoshihisa Fujino

**Affiliations:** 1 Department of Occupational Health Practice and Management, Institute of Industrial Ecological Sciences, University of Occupational and Environmental Health, Japan, 1-1 Iseigaoka, Yahatanishi-ku, Kitakyushu 807-8555, Japan; 2 Department of Work Systems and Health, Institute of Industrial Ecological Sciences, University of Occupational and Environmental Health, Japan; 3 Department of Mental Health, Institute of Industrial Ecological Sciences, University of Occupational and Environmental Health, Japan, 1-1 Iseigaoka, Yahatanishi-ku, Kitakyushu 807-8555, Japan; 4 Department of Disaster Occupational Health Center, Institute of Industrial Ecological Sciences, University of Occupational and Environmental Health, Japan, 1-1 Iseigaoka, Yahatanishi-ku, Kitakyushu 807-8555, Japan; 5 Department of Environmental Health, School of Medicine, University of Occupational and Environmental Health, Japan, 1-1 Iseigaoka, Yahatanishi-ku, Kitakyushu 807-8555, Japan; 6 Department of Preventive Medicine and Community Health, School of Medicine, University of Occupational and Environmental Health, Japan, 1-1 Iseigaoka, Yahatanishi-ku, Kitakyushu 807-8555, Japan; 7 Department of Environmental Epidemiology, Institute of Industrial Ecological Sciences, University of Occupational and Environmental Health, Japan, 1-1 Iseigaoka, Yahatanishi-ku, Kitakyushu 807-8555, Japan

**Keywords:** COVID-19, Vaccine hesitancy, Equitable allocation, Workplace vaccination, Company size, Socioeconomic factors

## Abstract

**Background:**

Vaccination is considered the most effective control measure against COVID-19. Vaccine hesitancy and equitable vaccine allocation are important challenges to disseminating developed vaccines. To promote COVID-19 vaccination coverage, the government of Japan established the workplace vaccination program. However, while it appears that the program was effective in overcoming vaccine hesitancy, the program may have hindered the equitable allocation of vaccines because it mainly focused on employees of large companies. We investigated the relationship between company size and COVID-19 vaccination completion status of employees and the impact of the workplace vaccination program on this relationship.

**Methods:**

We conducted an internet-based prospective cohort study from December 2020 (baseline) to December 2021. The data were collected using a self-administered questionnaire survey. Briefly, 27,036 workers completed the questionnaire at baseline and 18,560 at follow-up. After excluding ineligible respondents, we finally analyzed the data from 15,829 participants. At baseline, the participants were asked about the size of the company they worked for, and at follow-up they were asked about the month in which they received their second COVID-19 vaccine dose and the availability of a company-arranged vaccination opportunity.

**Results:**

In each month throughout the observation period, the odds of having received a second COVID-19 vaccine dose were significantly lower for small-company employees than for large-company employees in the sex- and age-adjusted model. This difference decreased after adjusting for socioeconomic factors, and there was no significant difference after adjusting for the availability of a company-arranged vaccination opportunity.

**Conclusions:**

The workplace vaccination program implemented in Japan to control the COVID-19 pandemic may have been effective in overcoming vaccine hesitancy in workers; however, it may have caused an inequitable allocation of vaccines between companies of different sizes. Because people who worked for small companies were less likely to be vaccinated, it will be necessary to enhance support of vaccination for this population in the event of future infectious disease outbreaks.

**Trial registration:**

Not applicable.

## Introduction

Vaccination programs are underway worldwide because vaccination is the most effective measure to control the coronavirus disease 2019 (COVID-19) pandemic, which was declared by the World Health Organization (WHO) in March 2020. Since the outbreak of COVID-19 in China in December 2019 [1], various types of vaccines have been developed in a short period of time [2]. Some of these are mRNA vaccines, representing a new type of vaccine technology [3].

Disseminating vaccines presents many challenges, among which vaccine hesitancy and equitable allocation are prominent. Vaccine hesitancy, defined as the “delay in acceptance or refusal of vaccination despite the availability of vaccination services” is considered a major public health challenge in infectious disease control because it delays vaccination of the population and inhibits the acquisition of herd immunity [4]. Various factors, including socioeconomic [5] and psychological factors [4], have been found to contribute to people’s vaccine hesitancy. Such factors have also been examined in the context of COVID-19 vaccination [6, 7]. The equitable allocation of vaccines is based on maintaining equity in the order of vaccination according to risk regardless of social status, for example by starting with healthcare workers and those at higher risk of serious illness [8].

In Japan, the majority of the population had some level of initial vaccine hesitancy to receive a COVID-19 vaccine [9, 10]. Nevertheless, by the end of December 2021, approximately 80% of the population had received two vaccine doses [11]. In Japan, COVID-19 vaccination efforts began on February 17, 2021 using two mRNA vaccines: one from Pfizer Inc. and one from Moderna Inc. In consideration of equitable vaccine allocation, the vaccination of healthcare workers was followed by the vaccination of older adults [12]. Thereafter, vaccination progressed through the general population in stages according to age.

An aspect of COVID-19 vaccination in Japan has been the availability of vaccination at workplaces in addition to community settings provided by municipalities and clinics [12]. Compared with other developed countries, the start of the vaccination program was delayed in Japan. To make up for this delay, the government appointed a minister to be in charge and set a goal of administering one million vaccinations per day. Part of the vaccination strategy was to implement the opportunity for workplace vaccination, which was conducted mainly by occupational health professionals such as occupational physicians and occupational health nurses. As a result, 9,654,000 people received their second vaccine dose through the workplace vaccination program, which started on June 21, 2021 [13]. Workplace vaccination, which provides a convenient vaccination opportunity, may have reduced vaccine hesitancy because several psychological and social factors can positively influence a person’s vaccination decision.

The workplace COVID-19 vaccination program in Japan, however, may have negatively affected the equitable allocation of vaccine doses. This program primarily targeted large companies, with a minimum of 2,000 doses to be delivered to a single location (i.e., an expected vaccination coverage of at least 1,000 persons [13]). Thus, there were barriers to its implementation in small and medium-sized companies. Therefore, company size may have affected the timing and coverage of employees receiving the second COVID-19 vaccine dose.

We hypothesized that while the workplace vaccination program facilitated COVID-19 vaccination, there was a size-dependent difference among companies in the timing of employees receiving the second vaccine dose and that this difference was influenced by the availability of a company-arranged vaccination opportunity. In a survey conducted in Japan during the COVID-19 pandemic, there were differences in the implementation of infection control measures and the opportunity to work remotely depending on the size of the company [14, 15]. Disparities in occupational health measures, such as workplace environmental and health measures, have arisen and depend on the size of the company. Such disparities have also been found in the establishment of COVID-19 countermeasures. Therefore, rather than the government’s workplace vaccination program ensuring vaccine equity, this program may have increased disparities in infection risk because of differences in the completion of COVID-19 vaccination based on company size.

We conducted a prospective cohort study to examine the relationship between company size and COVID-19 vaccination completion and the impact of the workplace vaccination program on this relationship, focusing on the period between July and December 2021, when the general population in Japan was receiving the second vaccine dose.

## Methods

### Study design and participants

This study was a part of the Collaborative Online Research on Novel-coronavirus Work Study (the CORoNa Work Study) and was conducted using a prospective cohort study design. The survey was commissioned to the internet survey company Cross Marketing Inc. (Tokyo, Japan), and the data were collected using a self-administered online questionnaire. All participants gave informed consent, and the study was approved by the ethics committee of the University of Occupational and Environmental Health, Japan (approval number: R2-079 and R3-006).

The baseline survey was conducted from December 22 to 25, 2020. The protocol for the baseline survey has been previously reported in detail [16]. The participants were aged 20–65 years and were employed at the time of the baseline survey (N = 33,087). Participants were included using cluster sampling by sex, age, region, and occupation. A total of 27,036 participants were included after excluding invalid response (n = 6,051).

**Fig. 1:**
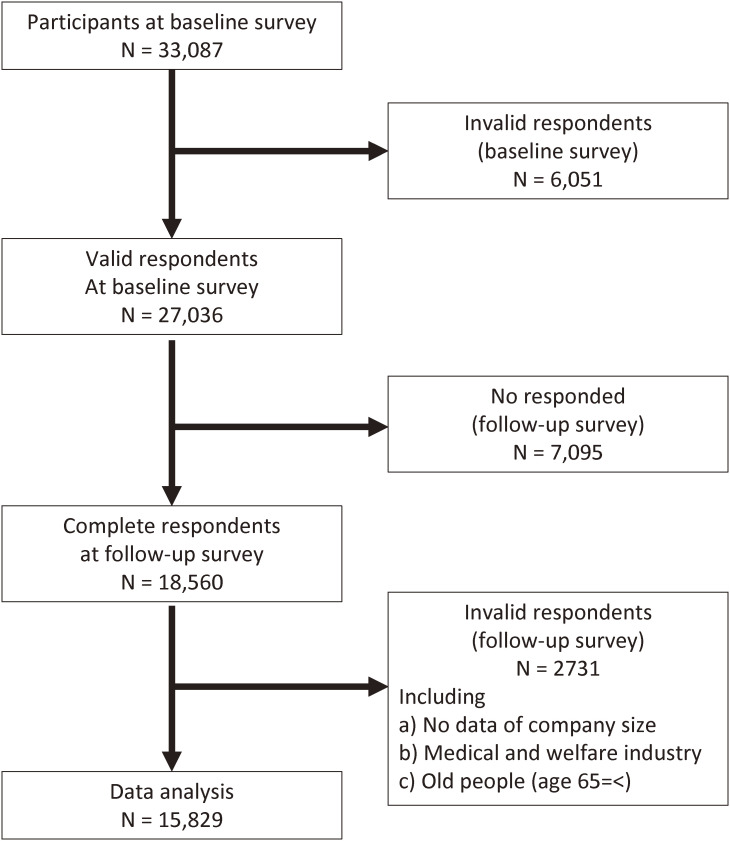
Flow diagram of the study participants

The follow-up survey was conducted from December 15 to 22, 2021, 1 year after baseline. A total of 18,560 participants responded to the survey. Among them, respondents were excluded if data on size of company they belonged to was unavailable (n = 160), they worked in the medical or welfare sectors (n = 2,341), or they were not working at the time of the follow-up survey (n = 230). Finally, 15,829 participants were included in the analysis. Figure 1 shows the flow diagram for this study.

### Second COVID-19 vaccination dose status

In the follow-up survey, we asked participants, “In what month did you receive the second COVID-19 vaccination?” Participants were requested to choose one of 12 options: the months of February 2021 through December 2021, or “have not received.” We then created a variable for completion status for each month after July. For example, completion by the end of September was defined as having received a second COVID-19 vaccine dose in any of the months from February through September. If a participant received the second vaccine dose in September, completion by July or August would be coded “no” but completion by September, October, November, and December would be coded “yes”.

### Company size

In the baseline survey, we asked participants, “How many employees are there at your company?” The participants could choose one of 10 options: 1 person (self-employed) or 2–4, 5–9, 10–29, 30–49, 50–99, 100–499, 500–999, 1000–9999, or 10,000 or more persons. We classified the responses into three categories: those who worked for small (1–49), medium-sized (50–999), or large (1,000 or more) companies. This classification was made because under the Industrial Safety and Health Act, the obligation to establish an occupational health management system differs depending on the size of the worksite [17]. Furthermore, the government-provided workplace vaccination program was eligible for locations that could vaccinate at least 1,000 people [13]. The size of workplace where participants worked was also asked and it may have had impact on vaccination coverage. We considered that companies, which had implemented workplace vaccination program, recognized the importance of equality among employees and had arranged for vaccination to be available to employees in as many workplaces as possible. Therefore, we used the size of company as an explanatory variable rather than that of workplace in this study.

### Company-arranged vaccination opportunity

In the follow-up survey, we asked participants, “Has your company arranged an opportunity to receive the COVID-19 vaccine at the workplace, whether or not you took advantage of the opportunity?” Participants could choose one of three response options: yes, no, or unknown. We regarded “yes” to indicate that the vaccination opportunity was arranged, and the other answers to indicate that this was not arranged.

### Assessment of covariates

Participant characteristics were collected at baseline. The covariates included socioeconomic factors, occupation, and industry. Age was classified into five groups: 20–29, 30–39, 40–49, 50–59, and 60–65 years. Annual household income was classified into five categories: <2.00 million Japanese yen (JPY), 2.00–3.99 million JPY, 4.00–5.99 million JPY, 6.00–7.99 million JPY, and 8.00 million JPY or greater. Educational background was classified into three categories: junior high or high school, vocational school or college, and university or graduate school. Marital status was classified into three categories: married, divorced or widowed, and unmarried. Occupation was classified into 10 categories: general employee; manager; executive manager; public employee, faculty member, or non-profit organization employee; temporary or contract employee; self-employed; small office/home office; agriculture, forestry, or fishing; professional occupation (e.g., lawyer, tax accountant); and other occupations. Participants could choose one of 22 options for their work industry, which was then classified into nine categories based on the International Standard Industrial Classification of All Economic Activities: manufacturing, public service, information and communication, wholesale and retail, food service, education and religion, finance and insurance, construction, and others.

### Statistical analysis

The odds ratios (ORs) for the association between company size and completion of the second COVID-19 vaccine dose were estimated using a multilevel logistic model nested in the prefecture of residence to account for regional variability. The multivariate model was adjusted for sex and age (Model 1) and additionally adjusted for annual household income, educational background, marital status, occupation, and industry (Model 2). Finally, the model was adjusted for company-arranged vaccination opportunity (Model 3).

A *p*-value of less than 0.05 was considered statistically significant. All analyses were conducted using Stata (Stata Statistical Software: Release 16; StataCorp LLC, College Station, TX, USA).

## Results

Table 1 shows the participant characteristics by company size. Of the 15,829 participants, 4,272 (27%) worked for a large company, 5,117 (32%) for a medium-sized company, and 6,440 (41%) for a small company. As the company size increased, the percentage of participants with a high annual household income and a high educational background level increased. Furthermore, as the company size increased, the opportunity for company-arranged vaccination increased: 56% for large companies, 35% for medium-sized companies, and 14% for small companies.

**Table 1:** Participant characteristics according to company size

		**Size of belonging company (Number of employees)**		
		**Large** **(1000 or more)**	**Medium** **(50–999)**	**Small** **(1–49)**
Total		4272	5117	6440

Age				
	20–29	185 (4.3%)	259 (5.1%)	173 (2.7%)
	30–39	611 (14.3%)	764 (14.9%)	769 (11.9%)
	40–49	1222 (28.6%)	1601 (31.3%)	1898 (29.5%)
	50–59	1755 (41.1%)	1929 (35.7%)	2511 (39.0%)
	60–65	499 (11.7%)	664 (13.0%)	1089 (16.9%)
Sex				
	Men	2781 (65.1%)	3172 (62.0%)	3785 (58.8%)
	Women	1491 (34.9%)	1945 (38.0%)	2665 (41.2%)
Annual household income (million JPY)				
	<2	128 (3.0)	226 (4.4%)	660 (10.3%)
	≥2 and <4	514 (12.0%)	990 (19.3%)	1594 (24.8%)
	≧4 and <6	828 (19.4%)	1316 (25.7%)	1610 (25.0%)
	≧6 and <8	997 (23.3%)	1068 (20.9%)	1105 (17.2%)
	≧8	1805 (42.3%)	1517 (29.6%)	1471 (22.8%)
Educational background				
	Junior high or high school	1018 (23.8%)	1426 (27.9%)	2117 (32.9%)
	Vocational school, junior college or technical school	613 (14.4%)	1003 (19.6%)	1575 (24.5%)
	University or graduate school	2641 (61.8%)	2688 (52.5%)	2748 (42.7%)
Marital status				
	Married	1244 (29.1%)	1704 (33.3%)	2296 (35.7%)
	Widowed/divorced	339 (7.9%)	432 (8.4%)	755 (11.7%)
	Never married	2689 (62.9%)	2981 (58.3%)	3389 (58.3%)
Occupation				
	General employee	2104 (49.3%)	2773 (54.2%)	2423 (37.6%)
	Manager	691 (16.2%)	753 (14.7%)	382 (5.9%)
	Executive manager	31 (0.7%)	107 (2.1%)	494 (7.7%)
	Public employee, faculty member, or non-profit organization employee	806 (18.9%)	606 (11.8%)	354 (5.5%)
	Temporary/contract employee	603 (14.1%)	810 (15.8%)	340 (5.3%)
	Independent business (commercial and industrial services)	9 (0.2%)	15 (0.3%)	1548 (24.0%)
	Small office/home office	3 (0.1%)	3 (0.1%)	270 (4.2%)
	Agricultural, forestry, and fishing industries	2 (0.0%)	3 (0.1%)	135 (2.1%)
	Professional occupation (lawyer, tax accountant, etc.)	11 (0.3%)	14 (0.3%)	140 (2.2%)
	Other occupation	12 (0.3%)	33 (0.6%)	354 (5.5%)
Industry				
	Manufacturing	1069 (25.0%)	1179 (23.0%)	755 (11.7%)
	Public service	638 (14.9%)	389 (7.6%)	203 (3.2%)
	Information and technology	312 (7.3%)	331 (6.5%)	287 (4.5%)
	Retail and wholesale	244 (5.7%)	355 (6.9%)	629 (9.8%)
	Eating/drinking	110 (2.6%)	182 (3.6%)	513 (8.0%)
	Education and religion	275 (6.4%)	417 (8.1%)	507 (7.9%)
	Finance	434 (10.2%)	213 (4.2%)	161 (2.5%)
	Construction	96 (2.3%)	139 (2.7%)	428 (6.7%)
	Other	1064 (25.6%)	1912 (37.4%)	2957 (45.9%)
Vaccination arranged by company				
	Yes	2383 (55.8%)	1780 (34.8%)	914 (14.2%)
	No	1889 (44.2%)	3337 (65.2%)	5526 (85.8%)
Month of 2nd COVID-19 vaccination				
	February-June	161 (3.0%)	205 (4.0%)	197 (3.1%)
	July	450 (10.5%)	467 (9.1%)	607 (9.4%)
	August	1267 (29.7%)	1354 (26.5%)	1550 (24.1%)
	September	941 (22.0%)	1140 (22.3%)	1380 (21.4%)
	October	774 (18.1%)	1063 (20.8%)	1212 (18.8%)
	November	240 (5.6%)	319 (6.2%)	420 (6.5%)
	December	21 (0.5%)	35 (0.7%)	40 (0.6%)
	Non-vaccinated	418 (9.8%)	534 (10.4%)	1034 (16.1%)

Table 2 shows the ORs for the association between company size and completion of the second COVID-19 dose by month. In the model adjusted only for age and sex (Model 1), participants who worked for a medium-sized company were significantly less likely to complete the second dose by August (OR = 0.87, 95% CI: 0.79–0.94, p = 0.001) and September (OR = 0.86, 95% CI: 0.78–0.93, p < 0.001) than those who worked for a large company. For small companies, the ORs decreased throughout the entire observation period, from July to December. In the model adjusted for the main socioeconomic factors (Model 2), the ORs for medium-sized and small companies tended to approach 1. For August and September, this tendency remained, but no significant difference was observed for the medium-sized companies. After adjusting for company-arranged vaccination opportunity (Model 3), the significant difference between small and large companies disappeared for the entire period analyzed. However, after October, participants who worked for medium-sized companies were significantly more likely to have received the second vaccine dose than those who worked for large companies (OR = 1.14, 95% CI: 1.01–1.28, p < 0.029). In each month throughout the observation period, those who had a company-arranged vaccination opportunity were significantly more likely to have received the second vaccine dose.

**Table 2:** Association between company size and completion of the second COVID-19 vaccine dose

**Second vaccination**		**model 1**			**model 2**			**model 3**		
**Comp. size**	**Cumulative** **coverage (%)**	**OD**	**95%CI**	**P value**	**OD**	**95%CI**	**P value**	**OD**	**95%CI**	**P value**
by July										
Large	14.3	Ref.			Ref.			Ref.		
Medium	13.1	0.93	0.82–1.05	0.229	0.98	0.86–1.10	0.691	1.06	0.94–1.21	0.330
Small	12.5	0.77	0.69–0.87	<0.001	0.79	0.69–0.90	0.002	0.94	0.82–1.07	0.350

Vaccination by comp.								1.56	1.40–1.74	<0.001
by August										
Large	44.0	Ref.			Ref.			Ref.		
Medium	39.6	0.87	0.79–0.94	0.001	0.92	0.84–1.00	0.065	1.05	0.96–1.15	0.277
Small	36.6	0.67	0.62–0.73	<0.001	0.76	0.69–0.83	<0.001	0.98	0.89–1.08	0.692

Vaccination by comp.								1.98	1.83–2.15	<0.001
by September										
Large	66.0	Ref.			Ref.			Ref.		
Medium	61.9	0.86	0.78–0.93	0.001	0.92	0.84–1.01	0.085	1.04	0.95–1.15	0.349
Small	58.0	0.64	0.59–0.70	<0.001	0.77	0.70–0.84	<0.001	0.97	0.88–1.07	0.541

Vaccination by comp.								1.90	1.76–2.07	<0.001
by October										
Large	84.1	Ref.			Ref.			Ref.		
Medium	82.6	0.92	0.82–1.02	0.127	1.00	0.89–1.12	0.971	1.14	1.01–1.28	0.029
Small	76.8	0.54	0.48–0.61	<0.001	0.75	0.67–0.84	<0.001	0.95	0.85–1.07	0.377

Vaccination by comp.								2.05	1.84–2.27	<0.001
by November										
Large	89.7	Ref.			Ref.			Ref.		
Medium	88.9	0.93	0.81–1.10	0.218	1.01	0.88–1.15	0.918	1.15	1.01–1.32	0.042
Small	83.3	0.54	0.48–0.61	<0.001	0.73	0.64–0.83	<0.001	0.94	0.82–1.08	0.362

Vaccination by comp.								2.14	1.88–2.43	<0.001
by December										
Large	90.2	Ref.			Ref.			Ref.		
Medium	89.6	0.94	0.82–1.07	0.329	1.02	0.89–1.17	0.759	1.17	1.02–1.35	0.029
Small	83.9	0.54	0.48–0.61	<0.001	0.73	0.64–0.83	<0.001	0.93	0.81–1.07	0.317

Vaccination by comp.								2.14	1.87–2.43	<0.001

model 1: adjusted for age and sex

model 2: model 1 + adjusted for annual household income, education, marital status, occupation and industry

model 3: model 2 + adjusted for company-arranged vaccination

Size of company: small (1–49); medium-sized (50–999); large (1000 or more)

Vaccination by comp.: vaccination arranged by company

## Discussion

This study showed that employees of smaller companies were less likely to have received a second COVID-19 vaccine dose. In the months after the start of the workplace vaccination program, the second dose completion rate of participants who worked for medium-sized companies was lower than that of those who worked for large companies, but this difference disappeared later in the observation period. The difference between large and medium-sized company employees could mostly be explained by differences in socioeconomic factor. The significant difference in completion rate between large company employees and small company employees remained throughout the observation period. Adjusting for the socioeconomic factors reduced the difference, but the significant difference remained. Therefore, the difference could partially be explained by differences in socioeconomic factors, but not completely. After adjusting for company-arranged vaccination opportunity, the difference in second dose completion rate between large and small companies disappeared. Furthermore, medium-size companies had higher vaccine completion coverage than large companies in the latter half of the observation period.

The smooth promotion of mass vaccination with pandemic vaccines is influenced by both supply- and demand-side factors. Supply-side factors include securing and transporting vaccines and developing a vaccination system, while demand-side factors are influenced by the public’s willingness to be vaccinated. In Japan, two types of mRNA vaccines and one type of virus-vector vaccine were approved for use, and two of these mRNA vaccines were used. Of these, the vaccine from Pfizer Inc. was used for vaccination of healthcare workers and for vaccination of the elderly and general population by local governments using local healthcare resources [12]. However, with the hit of the second wave of infection spread period in mid-June, the vaccine manufactured by Moderna Inc. was used to speed up the vaccination process, which included large-scale mass vaccination by medical personnel of the Self-Defense Forces [18], and workplace vaccination by medical personnel belonging to companies such as occupational physicians [13]. Workplace vaccination program, the subject of this study, was positioned as a supplemental route to vaccine supply through municipal vaccination. However, the contribution of the program to vaccination for working population was significant as more than half of the participants working in large companies and about one third of total participants had an opportunity to receive COVID-19 vaccine at the workplace in this study.

Regarding the demand side, there were concerns about the safety of newly developed vaccines in particular, and the presence of vaccine hesitancy owing to a lack of trust in vaccination and other factors has been a challenge to achieving herd immunity through vaccination. Socioeconomic factors have been found to affect vaccination intention and uptake of other vaccines, such as the seasonal influenza vaccine [19] and the H1N1 vaccine [20]. The effects of socioeconomic factors on vaccination intention for the COVID-19 vaccine have also been examined [6, 7]. Studies have generally found a positive association between vaccine uptake and annual income and educational background, although some studies have shown inverse associations [21, 22]. Several studies have found differences in willingness to vaccinate depending on one’s occupation and industry [23–25]. In the present study, after adjusting for these socioeconomic factors, the difference in vaccination completion rate between employees of medium and large companies disappeared, and the difference between employees of small and large companies became smaller. These findings suggest that socioeconomic factors affect the association between COVID-19 vaccination and company size in Japan. The differences by socioeconomic factors were also observed in an analysis of vaccination intentions conducted in December 2020 [10]. Therefore, the fact that the socioeconomic factors influenced vaccine uptake, despite the detailed vaccination regime in each region and free vaccination, is a finding that should be taken into account in future pandemic vaccination programs.

Workplace vaccination also affected demand-side factors. In the present study, it was observed that participants who had a company-arranged vaccination opportunity were significantly more likely to have received the second vaccine dose, and after adjusting for the presence of a company-arranged vaccination opportunity, no significant difference in the second dose completion rate was found between employees of small and large companies for all months. These results suggests that the government’s implementation of the workplace vaccination program had a positive impact on the vaccination acceptance of employees who worked for companies that participated in the program. The company-arranged vaccination opportunities may have decreased vaccine hesitancy and increased vaccination coverage. To evaluate the psychosocial factors influencing vaccine hesitancy, in 2011, the WHO Strategic Advisory Group of Experts proposed the “3C” model [4], which stands for “Confidence”, “Convenience”, and “Complacency.” German researchers subsequently proposed the “5C” model, substituting “Constraints” for “Convenience” and adding “Calculation” and “Collective responsibility” [26]. In the “5C” model, the “Confidence” includes trust in the vaccine provider, the company to which participants belong in this study. So, company-arranged vaccination opportunities are thought to increase people’s confidence in a vaccine, and the availability of the vaccine at or near their workplace increases its convenience. In addition, social environmental factors have been suggested to affect one’s vaccine intention [27, 28]. Previous studies on seasonal influenza vaccination in the U.S. have reported that workplace vaccination practices and recommendations are associated with higher vaccination coverage [29]. The workplace vaccination program facilitated employees’ vaccination behavior to be shared among coworkers and supervisors, which may have had a direct impact on social environmental factors, such as the social norm, herding effect and peer pressure. Among them, peer pressure is often considered an undesirable influence factor, but it is difficult to separate it from desirable social environmental factors.

The influence of socioeconomic factors and company-arranged vaccination opportunities on vaccination coverage has implications for the equitable allocation of vaccines. In the workplace vaccination program, the government invited companies that wished to implement the program on the premise that at least 1,000 people could be vaccinated at a single location [13]. Multiple small companies could apply if they could jointly secure more than 1,000 people willing to be vaccinated. However, because it was necessary to arrange venues and medical personnel for the vaccination event and to coordinate costs, program utilization may vary greatly depending on company size. During the COVID-19 pandemic in Japan, there were marked differences among companies of different sizes in the implementation of remote work and infection control measures [14, 15]. So, the size of the company may affect the risk of COVID-19 infection in the workplace. Nevertheless, the workplace vaccination programs led by the Japanese government have affected differences in vaccination coverage by the size of the company to which they belong, and it may have contributed to health disparities. Therefore, the pros and cons of a workplace vaccination program and the methods used to realize it warrant further discussion to ensure a more equitable implementation in future infectious disease outbreaks.

It is unclear why there was significantly higher vaccination completion among participants who worked for medium-sized companies compared with those who worked for large companies after October 2021 in the model adjusted for both socioeconomic factors and company-arranged vaccination opportunity. One possible explanation is that many employees of large companies were located in offices other than the headquarters and therefore had difficulty accessing the company-arranged vaccination opportunity. Another possibility is that, although COVID-19 vaccination was voluntary, medium-sized companies are often in a weaker business position than larger companies, and therefore they may have been more influenced by pressure from clients to vaccinate their employees in order to continue doing business.

This study had several limitations. First, the survey was conducted via the internet, so generalizations should be made with caution. For example, online panelists may be obtained information related to COVID-19 mainly through internet, and the main information source of participants may affect the vaccine acceptance [30]. However, we attempted to reduce any bias by using cluster sampling with stratification by sex, region and job type. Second, the study was likely affected by recall bias. The earlier vaccination was completed, the more time had elapsed by the time of the survey, which may have caused recall bias. However, since such effects are not expected to be related to the company size, their impact on this study will be small. Third, the timing of the follow-up survey might have affected the responses to the question of vaccination status in the last month, December. If a person received their second vaccine dose in the last week of December (after filling out the follow-up survey), they may have answered “unvaccinated” when asked about their vaccination status in the follow-up survey. However, the impact of this situation was likely small because second-dose vaccination was nearly complete in both the community and workplace programs by the end of November, and less than 1% of the respondents received their second vaccine dose in December. Fifth, it is possible that the size of the worksite, rather than the size of the company, had an impact. We collected both data of company size and worksite size from each participant, of which we used company size as an explanatory variable in this study because we believe that companies tended to arrange for vaccination to be available to employees in as many workplaces as possible. Sixth, the location of worksites may differ depending on the size of the company and differences in infection status and available vaccination opportunities by location may have affected vaccination coverage. However, since the provision of COVID-19 vaccine has been carried out meticulously in all municipalities and we used a multilevel logistic model nested in the prefectures of residence in this study, we considered the effect to be small.

## Conclusion

During the period when COVID-19 vaccinations were being administered to the general population in Japan, the coverage of receiving a second COVID-19 vaccine dose was significantly lower for those who worked for small companies than for those who worked for large companies. This difference could mostly be explained by the availability of a vaccination opportunity arranged by the employer as well as socioeconomic factors. In the event of future infectious disease outbreaks, it will be necessary to enhance support of vaccination for the employees of small companies.

## Abbreviations

COVID-19: coronavirus diseases 2019
mRNA: messenger RNA
WHO: World Health Organization
JPN: Japanese yen
OR: odds ratio
CI: confidence interval

## Declarations

### Availability of data and material

Data available on request from authors.
